# The Treatment of the Intestinal Diseases of Infancy

**Published:** 1885-07

**Authors:** Charles G. Stockton

**Affiliations:** Professor of Materia Medica and Therapeutics, Niagara University; 371 Porter Ave., Buffalo, N. Y.


### The Treatment of the Intestinal Diseases of Infancy.

By Charles G. Stockton, M.D.,
Professor of Materia Medica and Therapeutics, Niagara University.

Our honored President, who has a place for this society near his heart, selected the title of this paper, and that is authority enough to excuse me for writing upon an old subject. Yet it is one constantly before us, and, with every heated term, assuming such importance that doctors and societies, of doctors feel it their bounden duty to add their mite of experience to the end that the terrible infant mortality may be lightened.

The time has passed for priest, parent and practitioner to exclaim: "Submit, submit! It is the interposition of Providence." We feel that here is a waste of life, a loss of energy, a prolongation of the probation of the race. And in this spirit I shall expect a more excellent discussion to-night than the merit of the paper could of itself provoke.

A short article, in which some medical man compasses the entire subject in a few pages, setting forth the causes, the prevention and the cure of an affection, according to the writer's ideas, is common. I purpose following a part of the common plan, that is, to express briefly my views on the subject of infantile gastro-enteritis without any attempt at collaborating what has been written by others. We have to consider, in the first place, the alimentary tract of the very young, the functions of the various organs in this tract, and we have to remember that these organs are in a state of development; that they gain in strength, of function, increase their repertoire, as it were, as the child gains in age, but not always in proper ratio to the gain in age; and upon this I wish to lay particular stress. The digestive organs of a child three months old may be less developed than those of a healthful infant of three weeks. These organs are commissaries of the body. They have to supply aliment to children varying in race, temperament, diathesis and environment, not to speak of disease.

The aliment within reach is of two classes. First, the mother's milk.

Second, artificial food.

The mother's milk is generally wholesome, but not infrequently is unwholesome.

The artificial food is generally unwholesome, but may be made wholesome.

If, after this short survey, anyone asserts that the problem before us is simple, his powers of generalization surpass mine.

Let us now consider the organs of digestion more minutely. First, the mouth with its salivary and mucous glands, the secretions of which is alkaline in reaction, and, when introduced into the stomach, stimulates the flow of acid gastric juice. This secretion has also a ferment that converts amyloids, when the reaction is not acid. Second, the stomach, which secretes an acid juice, containing pepsine and hydrochloric acid, and which digests nitrogenous substances when the reaction is not alkaline. Third, the small intestines, whose alkaline secretions, supplemented by those with a like reaction from the pancreas and liver, digest sugars and fats, besides the leavings of starchy and albumunoid substances that have passed the pylorus. The flow of *succus-gastricus* is stimulated by the alkaline saliva, the presence of food, etc., but the *succus-entericus*, together with the secretion from the pancreas and liver, is stimulated by means of the acid gastric juice, etc., when the stomach's work is done.

I desire to emphasize the liver.

This organ, so large, so vascular, so active in the young child, is extremely susceptible to derangement from forces internal and external. The functions of these organs are under the control of a sensitive nervous apparatus. Let us keep this constantly before us.

The stomach and liver are most engaged in the digestive act during the early months, the salivary glands and pancreas lying comparatively dormant until later. It, therefore, follows that the nitrogenous, and, in a lesser degree, the saccharine food, are most likely to be provided for in the digestive tract of the infant, and that the starchy and fatty foods are unlikely to be assimilated until the child is further developed.

Having examined the physiological side of the question, let us follow the history of a case of gastro-enteritis.

The child nurses its mother. The milk has, besides water and salts, casein that coagulates in flocculi, some sugar and very little fat. It is very slightly alkaline or neutral in reaction, it is warm, and the child obtains it slowly, after a vigorous sucking, which stimulates the flow of alkaline saliva; and this, descending to the stomach, excites the flow of acid gastric juice, and this, in turn, the stomach having played its role, passes the pyloric ring in such a just and proper degree of acidity as to stimulate a yield of intestinal digestive fluids. If it were too feebly acid, the stimulation would be too little; if excessively acid, the intestinal apparatus would become over stimulated—irritated.

A proper developed child nursing a gentle and wholesome mother, goes on nursing and growing, never has colic or diarrhœa, and is a sight to warm a bachelor into a realization of his self-neglect.

But the infant whose history we are pursuing is deprived of its mother's breasts when two weeks old. Resort must now be had to artificial food.

This is a civilized community; so I will tone down the picture and suppose that an intelligent physician advises the child to be fed upon cow's milk, properly diluted and sweetened, to be taken through a rubber nipple.

The milk from a fairly fed cow is a fairly long time in transportation, and reaches the child with the lactic acid fermentation fairly begun. At the end of twenty-four hours, when the baby has its last feed from this milking, acetic acid fermentation is beginning. The baby receives the milk through a rubber nipple having too large an opening, and so without the effort of sucking. It reaches the stomach, not alkaline but acid in its reaction. This organ is not naturally stimulated, and it does not energetically secrete; yet it has to contend with a casein, coagulating rapidly into hard lumps, and with an over supply of fat. This passes the pylorus unaccompanied by its just proportion of hydrochloric acid. The bowels become inactive, the liver sluggish, the child has a coated tongue, the colic, sleepless nights, an anodyne, disturbed nervous system, a dose of oil—and then a temporary relief. After a few days, the poor stomach tires out, and its normal secretions are lessened. The nitrogenous, the saccharine and the oleaginous principles, instead of going in the way which nature has provided, are retained in the stomach until acetic and butyric acid fermentations are established, the gastric mucous membrane is irritated, and when at length the load passes into the duodenum, that organ, recently too inactive, is now excited, the liver becomes hyperæmic, bile is poured in the suffering duodenum, until it writhes and the baby writhes, and there is diarrhœa. The stomach revolts, and sour curds and whey with vitiated gastric juice are ejected. The alvine evacuations are acid.

The intelligent physician attempts to relieve these symptoms by administering alkalies. Lime-water is given with the milk, the amount of milk is lessened. This improves the complexion of things somewhat, and with the free use of castor oil to relieve the intestines of their mischievous contents, sometimes leads to convalescence. In this particular case, the improvement is limited. The child has appreciably wasted from innutrition and the loss of rest so necessary to healthy infancy. Then ensues fever and head symptoms, and vomiting. There is again intestinal inactivity, the few dejections being clay-colored, or there is diarrhœa of green, acid, offensive stools, with colic.

Then comes the inevitable prescription—calomel, gray powder, bismuth, Dover's powder, bromides, chloral, etc., etc.

Meanwhile, the intelligent practitioner is struggling with the question of diet. Evidently, there must be a change. He bethinks himself of condensed milk—and to that particular brand put up in Switzerland, where the cows enjoy forced quietude and where the scenery is sublime. Anything ought to thrive on such milk, but this child is obstinate and grows worse. Despite the lime-water, the excess of sugar ferments, or, if it reaches the circulation via the disordered liver, it is imperfectly oxidised and the baby has lithæmia.

The amount of lime-water is increased—and so is the vomiting; while the curds, like Banquo's ghost, will not down.

The intelligent practitioner perceives that milk must be withdrawn. The child is fed for days upon albumen water, gelatine water, or gluten water, and improves. But at length his little organs cry out for their natural pabulum, which the whites of eggs cannot provide, and the persecuted stomach will retain it no longer. Beef tea, beef peptones are resorted to. Good for one day only. A second intelligent practitioner is called in consultation, and appears to be surprised that milk, in combination with oatmeal, or barley, or biscuit food, was not given in the beginning, and quotes Dr. Jacobi. But the attendant remarks that he has thoroughly tried that plan and with frequent failures; besides, he is satisfied that this child will not digest milk in any form, apparently not knowing what it needs. Several prepared foods are discussed—and now they are upon dangerous ground. They recognize the impracticability of requiring an infant duodenum to digest starch, for it can rarely accomplish it at the best, and even when assimilated imperfectly, the infant is illy nourished, anæmic, a fit prey for any disease, a victim for any disorder. After due deliberation, Liebig's malt food is fortunately determined upon. In this food the starch is, by the action of malt, mostly converted into dexterine and maltose, and is a very acceptable nutriment to many children. In this case, however, it is not well tolerated and only imperfectly assimilated, owing to the state erythism into which the digestive tract has been driven by means of a disturbing diet and innutrition.

The subject of our history has now reached a most critical epoch in its career. Its tiny skeleton almost may be seen through its withered, skinny, clammy covering. The pinched face, with sunken eyes and shrunken lips,—the cranium with fontanels depressed and with bones that seem let loose by their softening cartilages, the puckered, retracted little belly, all tell of the waste that has been going on, and the baby's piteous wail will not deny it.

What is to be done with such a case? Some one replies, "Transplant it to the seaside or to the mountains." But in this instance such a removal is impracticable and, possibly, in the child's feeble condition, inadvisable. Is there anything else to be done at home? is the question that we are called upon to answer. In this history, I have endeavored to depict a typical case from a class which, at the age of six weeks or under, usually terminate fatally, and, in the discussion, pray remember that I am speaking of the very early weeks. "Almost one-half of the infant dead, before the end of the first year, die before they are one month old" (Jacobi).

I would propose this treatment:

Procure a pint of fresh, pure cow's milk, and prepare from it a whey, by mixing pure pepsine, grains five to ten, or a little infusion of rennet with the milk, bringing it to a temperature of 90° F., and when the curd appears, strain it, pressing out all that will flow. This whey should be placed immediately upon ice, and be prepared at least twice in twenty-four hours. Let the baby have one-fourth drop of the wine of ipecac, with five drops of lime water, or a little soda, properly diluted. In a few moments, let it nurse, through a bottle, an ounce of warmed and slightly sweetened whey. Repeat this as the stomach will permit, increasing the quantity with each feed, if admissible. Let the bowels be kept open by means of castor oil—the tasteless London oil is the best. Inunctions with cod-liver oil or a warm milk-bath may be employed. The child should be removed into the open air, but its body temperature carefully regulated by wraps and artificial heat.

After the lapse of one or two days, if the patient retains the whey, a feed of milk should be tried, peptonized after the following rule: Dissolve from three to five grains of pure pepsine in a pint of perfectly fresh, warm milk. (The milk from some cows requires more pepsine than from others.) Raise the temperature of the mixture to 90° F. Watch it constantly. In a few minutes, at the first sight of curding, remove and strain through a coarse linen, squeezing out the last drops of milk, and leaving a small residue in the strainer cloth.

This milk, if kept upon ice, will remain fit for use for twelve hours. It is partially peptonized milk. It should be diluted a little before feeding. It is slightly acid in reaction.

I should add the fraction of a drop of the tincture of nux vomica to the medicine already prescribed, giving it every two hours, and feeding the baby as often. If this milk is properly peptonized, it will be retained, and the child will smile at you in the morning. It requires some patience on the part of the nurse to learn the art of stopping the peptonization just at the right point. If you carried too far, she has whey instead of milk, and "Love's labor lost." No matter how troublesome it may be to prepare, milk thus treated will save life when nothing else will, or I greatly err in numerous observations.

After the lapse of days, the progress before noted often comes to a halt. The food doesn't seem to be agreeing well, and another change must be made. Hitherto, the food taken has been slightly acid, and it would seem reasonable to replace it with that which is slightly alkaline. Practically, this is found to be sound reasoning, and it is well to make the change before a retrograde action begins.

Still let the patient have peptonized milk, but prepare it with the extract of pancreas and a very little soda. It will disappoint you if too much is used. The original formula of the manufacturers of extractum pancreatis, Fairchild Bros. and Foster, was fifteen grains of soda to five grains of extract. This combination will be tolerated for a certain period of time, but not by infants with digestion greatly deranged. Five grains each of the extract and the soda to a pint of milk is about right, and, if you choose, the soda may be omitted entirely. At present, let us employ the small amount of soda, and keep the milk in a warm place until the bitter taste of the peptonate is just perceptible. Then place the milk on ice, and feed well diluted and slightly sweetened. In the place of the lime water in the mixture of nux vomica and ipecac, use half a drop of diluted hydrochloric acid; and, in place of giving it before, give it fifteen minutes after feeding.

I should expect the patient to thrive on this diet and treatment for a fortnight, and if evidence of indigestion should appear, return to the other form of peptonized milk.

The history above narrated, with the details of treatment and diet, is not supposible, but is an abstract from clinical experience.

Several years since, when summoned to care for an infant with intestinal disturbance and artificially fed, I expected it would die; and in nothing did my foresight prove more reliable. But now such cases so commonly recover, and it is so great a satisfaction to behold that the helpless little creatures grow in vigor, that there is compensation for all the time and care spent in digesting their dinners for them. As time and development advance, the baby may be successfully nourished on pure milk and water, and, later, farinaceous matter may be added. The diastatic properties of malt extract are at length understood by the profession in the treatment of disturbances in older patients, yet I do not see these well-known principles exercised in treating indigestion in young children. Before a child can successfully assimilate starchy food, it will flourish upon the same if it be mixed with the extract of malt. By this I mean that when bread and milk disagrees with and retards, bread and milk, in which a little malt extract is dissolved, favors comfortable digestion.

If people will persist in feeding infants on oatmeal, Imperial granum, *et hoc genus omne*, in the form of pap, let a little malt extract be used to sweeten the mixture.

It would be impossible, within the short space at my command this evening, to cover all the phases of the intestinal diseases of children. While I have really examined—yet not exhausted—a single type of these diseases, this may serve to bring out the central truths that bear upon allied conditions.

The child is undeveloped, is in a state of growth; some organs are occasionally behind their neighbors in the process; and the structures are yet so embryonic that much rest is needed for their repair.

Nature's food is adequate, and deviation from it perilous. In substituting, let us imitate nature; and to succeed, let us study physiology rather than advertisements.

Organic chemistry has not attained the secret of transforming cereals into breast-milk, and the philosopher's stone is undiscovered.

Physiology is the law of common practice. A respite may be granted, a ransom may be received, but lawlessness is always punishable.

I affirm with confidence that the methods of using milk set forth in this paper are physiological, and in compliance with the well-known law that an alkali excites an acid, and an acid excites an alkaline secretion. And not only this, these methods subserve another purpose, in assisting the stomach secretions on one occasion, and the intestinal secretions on another, thus inducing a fair division of rest and activity. Furthermore, I consider peptonization the greatest practical advance that has been made recently in the management of infantile diseases.

And after all, healthy infancy is largely a question of diatetics. What else remains has been epigrammatically stated by an eminent Italian, to be, "A clean skin, a sunny room, and a bottle of castor oil in the cupboard."

371 Porter Ave., Buffalo, N. Y.

